# William James Tivy

**Published:** 1910-09

**Authors:** 


### WILLIAM JAMES TIVY.

W. J. Tivy, F.R.C.P. Edin., F.R.C.S. Edin., L.M., was educated at Queen's College, Cork, his native city, also at Dublin, Edinburgh, and London. He was a Fellow of the Royal Society of Medicine, Fellow of the British Gynæcological Society, and of the Obstetrical Society of London. He was a member of our own Bristol Medico-Chirurgical Society, although he never took any active part in its work. His early years were spent in the neighbourhood of Hereford, but he settled in Clifton many years ago, and had a considerable reputation, more particularly across the Severn. Since the year 1889 he had been Honorary Surgeon to the Bristol Naval Volunteers, and he had shown much interest in ambulance work. A friend writes of him as follows :—

" Whenever there was a competition on hand there was Dr. Tivy, watch in hand, sometimes as time-keeper; rifle in hand, sometimes at the range ; even tiller in hand, as coxswain of the boat; always prize list in hand, organising the prize distribution, or writing to prominent citizens for the assistance which has made the prize list a valuable incentive to emulation. Yet, with all this zeal and energy, there was a strong desire for self-effacement. He was strongly opposed to any idea of display or of attention being drawn to himself on public occasions.

" When on his death-bed he was giving directions as to his funeral he was very particular to request that he should not receive full naval honours. He expressed himself thus : ' Just the gun-carriage and the Union Jack and the necessary men, together with the boat's crew whom I coxed so badly last year.'

" It is certain that many of his patients will mourn his loss.

It is equally certain that the Bristol Division of the R.N.V.R. will feel the loss of a friend and a worker.

"Open-hearted, generous, and full of life are the expressions used by an officer of the division when speaking of him whom we laid to rest after a troublous life known only to himself."

He died at the age of 59, and his funeral was such as he desired—a gun-carriage drawn by the men in uniform. Many friends attended at the Seamen's Chapel, and a stretcher squad from the Clifton Division of the St. John Ambulance was in attendance.

He made several contributions to medical literatuie, a list of which we append.

"Wood's Operation for Radical Cure of Hernia."—*Brit. M. J.*, 1878, ii, 559.

*Pathology and Treatment of Lateral Curvature of the Spine by the Poroplastic Jacket, Partial Recumbency, and Exercises.*—London: Hamilton, Adams and Co. 1885.

*Three Cases of Uterine Fibroids under Treatment by Apostoli's Electrical Method.—Ibid.*, 1888, i, 1376.

*Electrical Treatment of Uterine Fibroids, with Cases.* 1889.

*Heredity in Pulmonary Phthisis.—Ibid.*, 1901, ii, 437.

